# Identification of Two Novel *HOXB13* Germline Mutations in Portuguese Prostate Cancer Patients

**DOI:** 10.1371/journal.pone.0132728

**Published:** 2015-07-15

**Authors:** Sofia Maia, Marta Cardoso, Pedro Pinto, Manuela Pinheiro, Catarina Santos, Ana Peixoto, Maria José Bento, Jorge Oliveira, Rui Henrique, Carmen Jerónimo, Manuel R. Teixeira

**Affiliations:** 1 Department of Genetics and Cancer Genetics Group–CI-IPOP, Portuguese Oncology Institute–Porto, Porto, Portugal; 2 Department of Epidemiology, Portuguese Oncology Institute–Porto, Porto, Portugal; 3 North Region Cancer Registry, Portuguese Oncology Institute–Porto, Porto, Portugal; 4 Biomedical Sciences Institute (ICBAS), University of Porto–Porto, Portugal; 5 Department of Urology, Portuguese Oncology Institute–Porto, Porto, Portugal; 6 Cancer Biology and Epigenetics Group–CI-IPOP, Portuguese Oncology Institute–Porto, Porto, Portugal; 7 Department of Pathology, Portuguese Oncology Institute–Porto, Porto, Portugal; National Cancer Institute, National Institutes of Health, UNITED STATES

## Abstract

The *HOXB13* germline variant G84E (rs138213197) was recently described in men of European descent, with the highest prevalence in Northern Europe. The G84E mutation has not been found in patients of African or Asian ancestry, which may carry other *HOXB13* variants, indicating allelic heterogeneity depending on the population. In order to gain insight into the full scope of coding *HOXB13* mutations in Portuguese prostate cancer patients, we decided to sequence the entire coding region of the *HOXB13* gene in 462 early-onset or familial/hereditary cases. Additionally, we searched for somatic *HOXB13* mutations in 178 prostate carcinomas to evaluate their prevalence in prostate carcinogenesis. Three different patients were found to carry in their germline DNA two novel missense variants, which were not identified in 132 control subjects. Both variants are predicted to be deleterious by different *in silico* tools. No somatic mutations were found. These findings further support the hypothesis that different rare *HOXB13* mutations may be found in different ethnic groups. Detection of mutations predisposing to prostate cancer may require re-sequencing rather than genotyping, as appropriate to the population under investigation.

## Introduction

Prostate cancer (PrCa) is the most commonly diagnosed nonskin cancer among men in developed countries [1], where the cumulative risk by age 75 is estimated to be 8.8 [2]. The best-established risk factors for the development of PrCa are increasing age, African ancestry and family history of the disease [3], the latter being present in 10–20% of the patients [4]. Case-control and cohort studies have shown that the relative risk of PrCa increases substantially with increasing number of affected relatives and with decreasing age at onset [5] and that monozygotic twins have a fourfold increased concordance rate of PrCa compared to dizygotic twins [6].

Hereditary prostate cancer (HPC), which is estimated to account for 5–10% of all PrCa cases, refers to nuclear families with at least three cases of PrCa, families with PrCa in each of three generations in the paternal or maternal lineage, or a cluster of two relatives diagnosed before the age of 56, whereas aggregation of PrCa cases in a family that does not fulfill these criteria is classified as familial PrCa [7]. Several authors suggest that up to 43% of the patients diagnosed before the age of 56 may carry a high-risk allele [6,8], but the identification of highly penetrant genes in familial/hereditary PrCa has been more difficult than for other types of common cancer, namely those of the breast and colon. Although present in only 1–2% of all PrCa cases, germline mutations in the *BRCA2* gene were the first well-established genetic risk factor [9], conferring a 7.3 to 8.6-fold increased risk of developing the disease before the age of 65 when compared to non-carriers [10]. In early-onset PrCa, the prevalence of *BRCA2* mutations rises to 2.9%, conferring a 23-fold increased risk of developing the disease by the age of 56 years [11].

In the beginning of 2012, *HOXB13* was identified as a susceptibility gene for PrCa [12]. A rare, but recurrent germline variant [G84E, p.(Gly84Glu), c.251G>A, rs138213197] was reported among men of European descent and was associated with PrCa risk. Furthermore, the carrier frequency was higher in patients with early-onset (2.2%) or a positive family history of PrCa (2.2%) and highest in the group with both family history and early-onset disease (3.1%). The lowest mutation frequency was seen in patients with late-onset and nonfamilial PrCa (0.6%). Since this publication, several studies have confirmed the association between the G84E variant and PrCa risk [13–17], being responsible for up to 5% of familial/hereditary PrCa cases in men of European descent. Interestingly, the G84E mutation has not been found among PrCa patients of African or Asian ancestry [12,15,18], who instead show other *HOXB13* mutations, evidencing allelic heterogeneity in different ethnic backgrounds. The G84E variant is now considered to be a founder mutation that arose in Northern Europe, where its prevalence is highest [14,16,17], and *HOXB13* stands out as the most widely replicated susceptibility gene for PrCa uncovered to date.

Since most previous studies have genotyped only the G84E variant and limited data exist for southern European populations, we performed sequencing of the *HOXB13* coding region to evaluate the frequency of germline mutations in 462 early-onset or familial/hereditary PrCa cases. Furthermore, we evaluated the prevalence of somatic *HOXB13* mutations in PrCa by sequencing the *HOXB13* coding region of 178 prostate carcinomas.

## Materials and Methods

### Ethics Statement

This study was approved by the Institutional Ethics Committee of the Portuguese Oncology Institute-Porto (approval number: 38.010) and written consent was obtained from all participants.

### Early-onset and familial/hereditary PrCa

This study comprised a total of 462 index cases (HPC samples) from families with early-onset and/or familial/hereditary PrCa, which were selected based on one of the following criteria: 1) PrCa diagnosis before the age of 56 or 2) PrCa diagnosis at any age with family history of the disease (up to fourth degree relatives) and at least one family member (the proband or a relative) with PrCa before the age of 66. Most patients were invited to participate in a study with the main purpose of identifying germline mutations associated with inherited PrCa predisposition, having as starting point all living patients registered at the North Region Cancer Registry (RORENO) with a PrCa diagnosis before the age of 66, whereas a minority of the families had been referred for genetic counseling due to early-onset or family history. All but two patients (one from the United Kingdom and another from Angola) had at least one Portuguese ancestor. No systematic PrCa screening program exists in the population under study, only opportunistic screening is offered to men over 50 years of age. DNA was extracted from peripheral blood leukocytes using the MagNA Pure LC DNA Isolation Kit—Large Volume (Roche Diagnostics GmbH, Penzberg, Germany) and whenever DNA was available from more than one affected relative per family, the youngest at the time of diagnosis was considered as the index case.

All but two patients (one with prostatic basal cell carcinoma and another with carcinosarcoma of the prostate) had the histopathological diagnosis of prostate adenocarcinoma. The mean age at diagnosis of the index cases was 56.3 years (range: 36–79 years), with 52.4% of the patients diagnosed before the age of 56, 46.3% between ages 56 and 65 and only 1.3% after 65 years of age (with only one case after age 70). Regarding the inclusion criteria we had established for this study, 151 (32.7%) fulfilled the age criterion only, 220 patients (47.6%) fulfilled the family history criterion only, and 91 patients (19.7%) fulfilled both criteria. Of the total of 462 families studied, 74 fulfill the classical Hopkins criteria for hereditary PrCa [7]. Demographic, clinical and pathological characteristics are summarized in Table 1. Medical history was collected during medical appointments whenever possible or from medical records, and family history was self-reported.

**Table 1:** Demographic and clinicopathological characteristics of subjects with early-onset or familial/hereditary PrCa.

Parameter	No. (%)
**Age at diagnosis**	
≤55 years	242 (52.4%)
56–65 years	214 (46.3%)
>65 years	6 (1.3%)
**Family history**	
Yes	311 (67.3%)
No	151 (32.7%)
**Hopkins criteria**	
Yes	74 (16.0%)
No	388 (84.0%)
**Method of detection**	
Screening	329 (71.2%)
Symptoms	125 (27.1%)
Unknown	8 (1.7%)
**PSA at diagnosis (ng/ml)**	
<4.0	32 (6.9%)
4.0–9.9	291 (63.0%)
10–20	85 (18.4%)
>20	47 (10.2%)
Unknown	7 (1.5%)
**Gleason score**	
≤6	180 (39.0%)
7	211 (45.7%)
≥8	65 (14.1%)
Unknown	5 (1.1%)
N/A (basal cell carcinoma)	1 (0.2%)
**T-stage** *	
cT1	38 (8.2%)
cT2	70 (15.2%)
cT3	30 (6.5%)
cT4	6 (1.3%)
pT2	144 (31.2%)
pT3	153 (33.1%)
pT4	1 (0.2%)
Unknown	20 (4.3%)
**N-stage**	
N0	206 (44.6%)
N1	8 (1.7%)
Nx	248 (53.7%)
**M-stage**	
M0	326 (70.6%)
M1	7 (1.5%)
Mx	129 (27.9%)

N/A–not applicable

* Clinical staging is indicated for those patients for whom pathological staging was not available (n = 144).

### Tumor samples

We searched for somatic *HOXB13* mutations in 178 DNA samples extracted from prostate carcinomas from patients unselected for family history or age at onset, with clinically localized PrCa diagnosed and treated with radical prostatectomy at our institution between 2001 and 2006. Only 26 subjects from the prostatectomy case series studied for somatic mutations are shared with the 462 index cases studied for germline mutations. These tumors belong to a previously described consecutive series of 200 prostate carcinomas [19] from which good quality tumor DNA was available. All tissue specimens had been frozen immediately after surgery and stored at -80°C. Five-micron thick sections were cut and stained for the identification of the areas of PrCa and then the tissue block was trimmed to maximize the yield of target cells (>70% of tumor cells). Subsequently, an average of fifty 12-micron thick sections were cut and every 5^th^ section was stained to ensure an uniform percentage of target cells. DNA was extracted from tumor tissue by the phenol/chloroform method using phase-lock gel (5 PRIME, Hamburg, Germany). The mean age at diagnosis was 63.4 years (range: 49–74 years) and the pathological stage was pT2 and pT3 in 54.6% and 45.4% of the cases, respectively.

### *HOXB13* sequencing

For mutation screening of the entire *HOXB13* coding region, specific primer pairs (S1 Table) were designed using the Primer-BLAST design tool from the National Center for Biotechnology Information (NCBI) [20]. A touchdown PCR was performed with an initial step of denaturation at 97°C for 10 minutes, followed by four sets of cycles (6 + 6 + 6 + 22) with denaturation at 97°C for 1 minute and extension at 72°C for 2 minutes. Annealing occurred at 64°C for 1 minute in the first set of cycles, and at 62°C, 60°C and 56°C for 30 seconds for the subsequent sets. The reaction ended with a final extension step at 72°C for 10 minutes. Sanger sequencing was performed using BigDye Terminator v3.1 Cycle Sequencing Kit (Applied Biosystems, Carlsbad, CA), following the manufacturer’s instructions. The products were run on a 3500 Genetic Analyzer (Applied Biosystems) and sequences were compared with the *HOXB13* NCBI reference sequence (NM_006361.5) using the Mutation Surveyor V4.0.7 software (Softgenetics, State College, PA, USA).

### Genotyping of controls

As control subjects, we used 132 male blood donors (mean age 56.8 years; SD±5.1 years) from the Portuguese Oncology Institute of Porto with no personal history of cancer at the time of blood collection. The non-synonymous substitutions detected in the PrCa patients were screened in the controls using KASPar SNP genotyping (KBioscience, Herts, UK) on a Roche LightCycler 480 Real-Time PCR System, according to manufacturer’s instructions. KASPar assay primers (S1 Table) were designed using the Primer-BLAST design tool from NCBI [20]. Data were analyzed in the LightCycler 480 Software 1.5.0.

### *In silico* analysis of variants

In order to predict the functional impact of the identified variants, we used several bioinformatic tools. Missense variants were evaluated by PolyPhen2 [21], PROVEAN [22], SIFT [23], MutationTaster [24], MutPred [25], MutationAssessor [26] and ESEfinder 3.0 [27]. PROVEAN, SIFT and ESEfinder 3.0 were also queried for synonymous variants and MutationTaster for synonymous and intronic variants. The PROVEAN interface was used to calculate both PROVEAN and SIFT scores. The Alamut Visual (version 2.6.1) software (Interactive Biosoftware, Rouen, France), which incorporates SpliceSiteFinder-like [28], MaxEntScan [29], NNSPLICE [30], GeneSplicer [31] and Human Splicing Finder [32], was used to assess intronic variants. Default thresholds were applied in all splice site prediction programs.

To verify the evolutionary conservation of the respective amino acid positions, multiple protein sequence alignment using human and orthologous species sequences was performed using Clustal W2 [33]. PhyloP [34] and Grantham [35] scores for missense variants were also determined by Alamut Visual.

### Haplotype analysis

Germline DNA samples of both patients (HPC311 and P308T) sharing the same *HOXB13* germline mutation were genotyped for the 11 microsatellite markers (6 localized in chromosome 17 and one for chromosome 2, 3, 5, 9 and 13) indicated in S2 Table. All 11 markers were assayed by PCR using fluorescently end-labeled primers (S2 Table) and capillary electrophoresis was performed on an ABI PRISM 310 Genetic Analyser (Applied Biosystems, Foster City, CA).

## Results

### *HOXB13* variants

**Fig 1:**
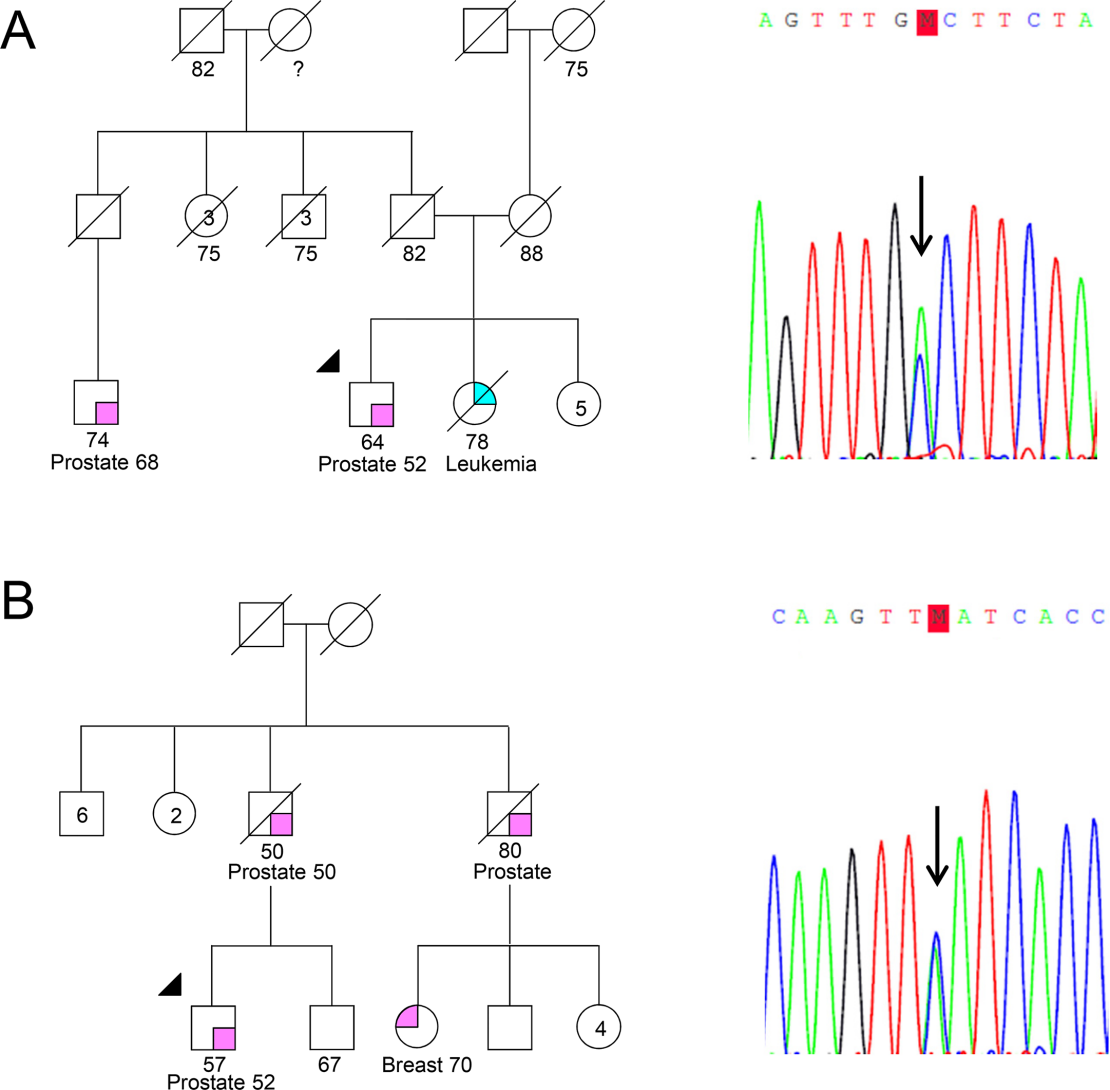
Pedigrees and Sanger sequencing electropherograms of patients from early-onset and familial/hereditary PrCa families. Pedigrees and electropherograms of patient HPC311 with the *HOXB13* p.(Ala128Asp), c.383C>A variant (A) and patient HPC169 with the *HOXB13* p.(Phe240Leu), c.720C>A variant (B). The index case and the position of each mutation are indicated by an arrow.

The G84E mutation was not found in any of the 462 patients with early-onset and/or familial/hereditary PrCa. However, four patients (samples HPC311, HPC169, HPC474 and HPC138) carried four different heterozygous variants, which, to our knowledge, have not yet been described in any published study or public database, namely the 1000 Genomes Project and the Exome Variant Server (Table 2). Subject HPC311 carries a transversion from a cytosine to an adenosine at position 383 (Fig 1), leading to an amino acid substitution from alanine to aspartic acid in codon 128 [c.383C>A, p.(Ala128Asp)]. A transversion from a cytosine to an adenosine at position 720 was found in subject HPC169 (Fig 1), leading to an amino acid substitution from a phenylalanine to a leucine in codon 240 [c.720C>A, p.(Phe240Leu)]. A synonymous substitution of a thymine by an adenosine at position 96 (c.96T>A) and an intronic variant (c.602-19T>G) were found in patients HPC474 and HPC138, respectively.

**Table 2:** Germline variants detected in prostate cancer patients.

Samples	Variant GRCh37 position	rs ID	cDNA change	Genotype	Protein change	1000G* MAF%	Exome Variant Server MAF%
HPC311 + P308T	17:46805573	N/A	c.383C>A	Het	p.(Ala128Asp)	Not reported	Not reported
HPC169	17:46804287	N/A	c.720C>A	Het	p.(Phe240Leu)	Not reported	Not reported
HPC474	17:46805860	N/A	c.96T>A	Het	p. (=)	Not reported	Not reported
HPC138	17:46804424	N/A	c.602-19T>G	Het	N/A	Not reported	Not reported
HPC2	17:46804414	rs148901331	c.602-9G>A	Het	N/A	IBS: 0.00% (0/214); GBR: 0.00% (0/182); ASW: 0.00% (0/122).	EA: 0.07% (6/8600); AA: 0% (0/4406); All: 0.05% (6/13006).
HPC374 + HPC461	17:46804124	rs371753257	c.*28C>A	Het	N/A	IBS: 0.00% (0/214); GBR: 0.00% (0/182); ASW: 0.00% (0/122).	EA: 0.03% (3/8600); AA: 0% (0/4406); All: 0.02% (3/13006).
HPC517	17:46805626	rs33993186	c.330C>A	Het	p. (=)	IBS: 0.00% (0/214); GBR: 0.00% (0/182); ASW: 1.64% (2/122).	EA: 0.16% (14/8600); AA: 2.36% (104/4406); All: 0.91% (118/13006).
multiple samples	17:46805590	rs8556	c.366C>T	Het/Hom	p. (=)	IBS: 13.08% (28/214); GBR: 12.64% (23/182); ASW: 25.41% (31/122).	EA: 13.21% (1136/8600); AA: 26.24% (1156/4406); All: 17.62% (2292/13006).
multiple samples	17:46805443	rs9900627	c.513T>C	Het/Hom	p. (=)	IBS: 9.81% (21/214); GBR: 10.44% (19/182); ASW: 10.66% (13/122).	EA: 9.60% (826/8600); AA: 10.74% (473/4406); All: 9.99% (1299/13006).

*1000 Genomes Project phase 3; AA–African-American; ASW–Americans of African Ancestry in Southwest USA; EA–European-American; GBR–British of England and Scotland; Het–Heterozygous; Hom–Homozygous; IBS–Iberian population in Spain; MAF–Minor allele frequency; N/A–Not applicable; p. (=) –protein has not been analyzed, but no change is expected.

Additionally, we found five other variants that are listed both in the 1000 Genomes Project and Exome Variant Server: three synonymous (c.330C>A, c.366C>T and c.513T>C), one intronic (c.602-9G>A) and one in the 3’ untranslated region (c.*28C>A). The reported allele frequency in the 1000 Genomes Project and Exome Variant Server of rs148901331 (c.602-9G>A) and rs371753257 (c.*28C>A) is lower than 1%. Variant rs33993186 (c.330C>A) seems to be more frequently found in individuals with African ancestry, although our patient (HPC 517) did not report African descent. The minor allele frequencies (MAF) for rs8556 (c.366C>T) and rs9900627 (c.513T>C) were 13.2% and 13.0%, respectively, in our samples, which is in concordance with existent data (Table 2).

**Fig 2:**
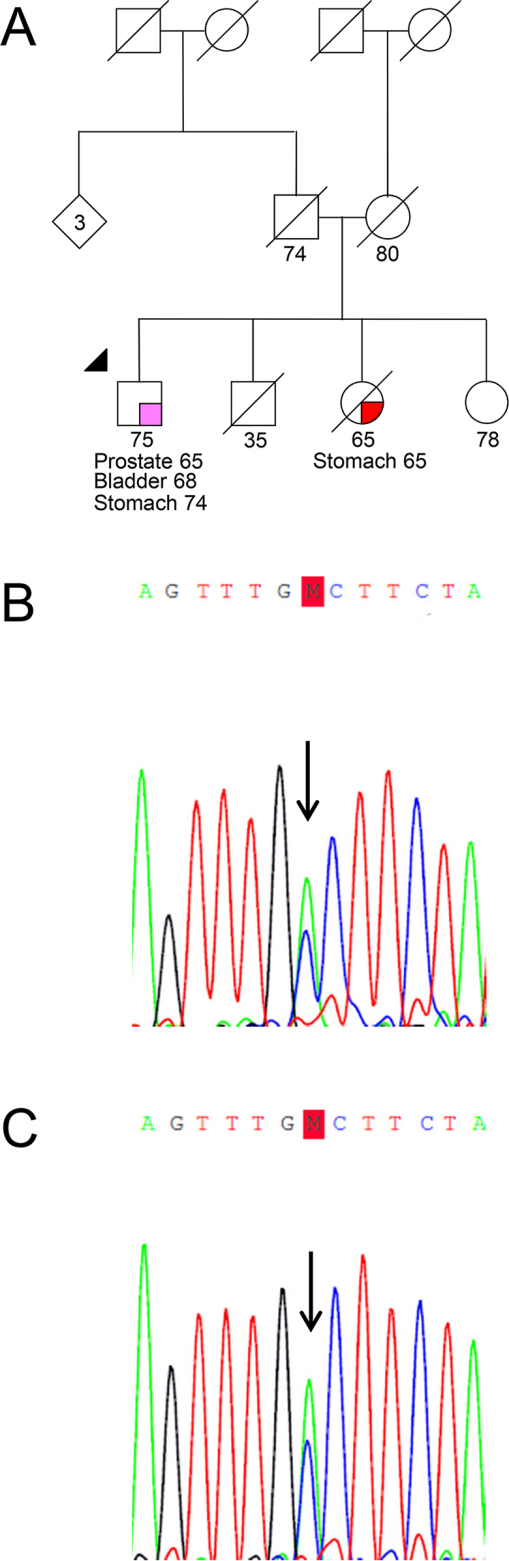
Pedigree and Sanger sequencing electropherograms of the patient from the prostate carcinoma series. Pedigree (A) and electropherograms of patient P308T obtained from tumor (B) and from blood (C) show the presence of the *HOXB13* c.383C>A, p.(Ala128Asp) variant in heterozygosity. The index case and the mutation are indicated by an arrow.

Regarding the tumor samples, the p.(Ala128Asp) variant was the only alteration found (patient P308T, in heterozygosity). As DNA extracted from peripheral blood from this patient was available, we were able to confirm that the missense alteration was present in the germline and was therefore not a somatic mutation (Fig 2).

Finally, we genotyped 264 alleles (132 control subjects) for the two non-synonymous missense variants using the KASPar SNP Genotyping System and none of the alterations were found.

### *In silico* analysis of variants

To explore the functional impact of the identified *HOXB13* variants, we used various bioinformatic tools. Variants rs8556 and rs9900627 were excluded from this analysis because of their high frequency. Regarding coding variants (Table 3), the missense variants p.(Ala128Asp) and p.(Phe240Leu) were predicted to be pathogenic by PolyPhen2, PROVEAN, SIFT and MutationTaster. The synonymous variants c.96T>A and c.330C>A were classified as disease-causing by MutationTaster, but neutral and tolerated by PROVEAN and SIFT, respectively. The probabilities of the missense variants being deleterious predicted by MutPred, the functional impact combined score predicted by MutationAssessor, and the predicted effects on exonic splicing enhancers given by ESEfinder 3.0 are shown in Table 3. Concerning noncoding variants (Table 4), all were categorized as polymorphisms by MutationTaster. As to the Alamut Visual splicing predictions, the NNSPLICE and GeneSplicer results suggest that the intronic variant c.602-19T>G might interfere with the recognition of the natural acceptor splice site, whereas SpliceSiteFinder-like and Human Splicing Finder predict no change between wild-type and mutated sequences. The results for the intronic variant c.602-9G>A were even more inconsistent, with NNSPLICE predicting a decrease and MaxEntScan an increase in the splicing efficiency (Table 4).

**Table 3:** *In silico* pathogenicity prediction of the coding *HOXB13* variants.

cDNA change, Protein change	PolyPhen2	Provean	SIFT	Mutation Taster (Probability values)	MutPred (Probability of deleterious mutation)	Mutation Assessor (FI score)	ESEfinder 3.0
c.383C>A, p.(Ala128Asp)	Probably damaging (1.000)	Deleterious (-4.27)	Damaging (0.000)	Disease causing (≈1.0)	0.239	Medium (2.525)	Weak alteration (SRSF2)
c.720C>A, p.(Phe240Leu)	Probably damaging (0.999)	Deleterious (-5.09)	Damaging (0.001)	Disease causing (≈1.0)	0.590	Medium (2.79)	ESE disruption (SRSF6)
c.96T>A, p. (=)	N/A	Neutral (0)	Tolerated (0.48)	Disease causing (≈1.0)	N/A	N/A	ESE creation (SRSF1, SRSF1:IgM-BRCA1 and SRSF5) and weak alteration (SRSF1:IgM-BRCA1)
c.330C>A, p. (=)	N/A	Neutral (0)	Tolerated (0.566)	Disease causing (≈1.0)	N/A	N/A	ESE disruption (SRSF1:IgM-BRCA1) and weak alteration (SRSF1, SRSF1:IgM-BRCA1, SRSF2 and SRSF6)

FI score–Functional impact combined score; N/A–not applicable; p. (=) –protein has not been analyzed, but no change is expected.

**Table 4:** *In silico* pathogenicity prediction of the noncoding *HOXB13* variants.

cDNA change	Mutation Taster (probability values)	SSF [0–100]	MaxEnt [1–16]	NNSPLICE [0–1]	GeneSplice [0–15]	HSF [0–100]
c.602-19T>G	Polymorphism (≈1.0)	= 75.18	6.89 → 6.71 (-2.5%)	0.69 → 0.46 (-34.1%)	10.89 → 9.32 (-14.4%)	= 82.05
c.602-9G>A	Polymorphism (≈1.0)	75.18 → 74.92 (-0.3%)	6.89 → 7.61 (+10.5%)	0.69 → 0.63 (-9.4%)	10.89 → 10.76 (-1.2%)	82.05 → 81.93 (-0.1%)
c.*28C>A	Polymorphism (≈1.0)	N/A	N/A	N/A	N/A	N/A

N/A–not applicable

**Fig 3:**
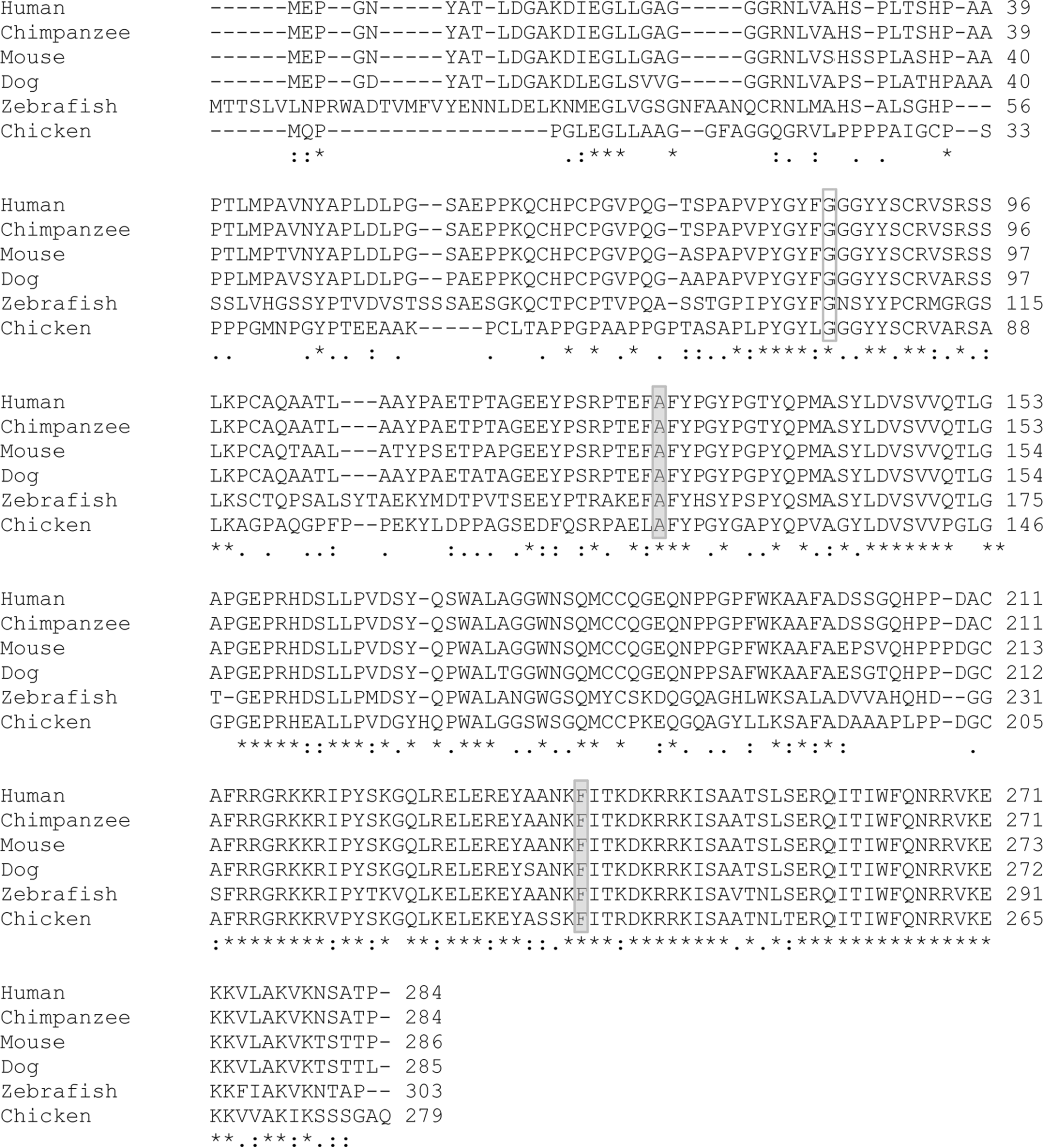
Clustal W2 alignment of the human HOXB13 protein and its orthologues in selected species. The amino acids residues predicted to be changed by both missense mutations identified in this study [p.(Ala128Asp) and p.(Phe240Leu)] are highlighted by grey shaded boxes and residue 84 predicted to be affected by the previously described G84E mutation is highlighted by a grey box.

In regard to the evolutionary nucleotide conservation, the p.(Ala128Asp) variant occurs in a highly conserved nucleotide (PhyloP score: 0.89), whereas the p.(Phe240Leu) alteration occurs in a nonconserved nucleotide (PhyloP score: -0.35). HOXB13 protein sequence alignment using Clustal W2 shows that both Alanine at position 128 and Phenylalanine at position 240 are fully conserved among the species indicated in Fig 3. The physicochemical difference between amino acids is moderate for the p.(Ala128Asp) variant (Grantham score: 126) and small for the p.(Phe240Leu) variant (Grantham score: 22).

### Clinicopathological characteristics of *HOXB13* mutation carriers

Patients HPC311 and HPC169 belong to our series of early-onset and/or familial/hereditary PrCa and they carry the c.383C>A, p.(Ala128Asp), and the c.720C>A, p.(Phe240Leu), mutations, respectively. Both have family history of PrCa (Fig 1) and were diagnosed at the age of 52, thus fulfilling not only the family history criterion, but also the age criterion we had established for this series. Patient HPC311 had a serum PSA at diagnosis of 6.2ng/ml, was initially treated with radical prostatectomy [Gleason score: 7 (3+4); TNM stage: pT3bN0M0] and four years later, because of rising PSA levels, he also received external radiotherapy. One of his sisters was diagnosed with leukemia. Patient HPC169 had a serum PSA at diagnosis of 4.06ng/ml, a biopsy Gleason score of 6 (3+3), a cT2NxM0 clinical stage, and he was treated with brachytherapy alone. He has a paternal cousin that was diagnosed with breast cancer at the age of 70.

Patient P308T belongs to the prostate carcinoma series and carries the same germline mutation found in patient HPC311 [c.383C>A; p.(Ala128Asp)]. Having been diagnosed with PrCa at the age of 65 and having no family history of PrCa (Fig 2), he did not fulfill any of the criteria we had established for the germline study. He had a serum PSA at diagnosis of 6.5ng/ml and was treated with radical prostatectomy [Gleason score: 6 (3+3); TNM stage: pT2cN0M0]. At the age of 68 he was also diagnosed with a high-grade papillary urothelial carcinoma of the bladder and six years later with a gastric adenocarcinoma. He has a deceased sister with the diagnosis of gastric cancer at the age of 65 years.

Given the fact that the two affected relatives of patient HPC169 were already deceased and that the affected relative of patient HPC311 is living abroad, it was not possible to perform segregation analysis in any of the affected relatives. Regarding other types of cancer, segregation analysis was not performed either because it would not be informative (late-onset breast cancer) or because the affected relatives were already deceased (Figs 1 and 2).

### Haplotype analysis

Genotyping of polymorphic microsatellite markers in patients P308T and HPC311 carrying the *HOXB13* c.383C>A germline mutation showed that, although we could not phase a haplotype for these two individuals (due to unavailability of relatives for testing), they share one allele in five of the six markers in 17q (where *HOXB13* is located), whereas the same consistent pattern was not observed for markers located in other chromosomes (S1 Fig; S2 Table). Neither genotypic nor pedigree data indicate that the two patients are closely related.

## Discussion

Since the first publication reporting *HOXB13* as a susceptibility gene for PrCa [12], several other authors have confirmed the association between the G84E variant and PrCa risk [13–16]. Although the reported frequency varies according to the studied population [14–17,36], the carrier frequency seems to be generally higher in patients with early-onset or a positive family history of PrCa and highest in the group with both family history and early-onset disease [12,16,17,37,38]. Several studies have also reported that the G84E variant is not found in men without European ancestry [16,39], but other *HOXB13* mutations have been reported in patients of African and Asian descent [12,15,18]. No systematic study of *HOXB13* mutations exists in PrCa patients from southern Europe; in fact, the only data available from populations from this region is the absence of the G84E variant in 183 men enrolled in the chemoprevention trial REDUCE, including 165 from Portugal, although it is unclear how many of these subsequently developed PrCa during follow-up [40].

**Fig 4:**
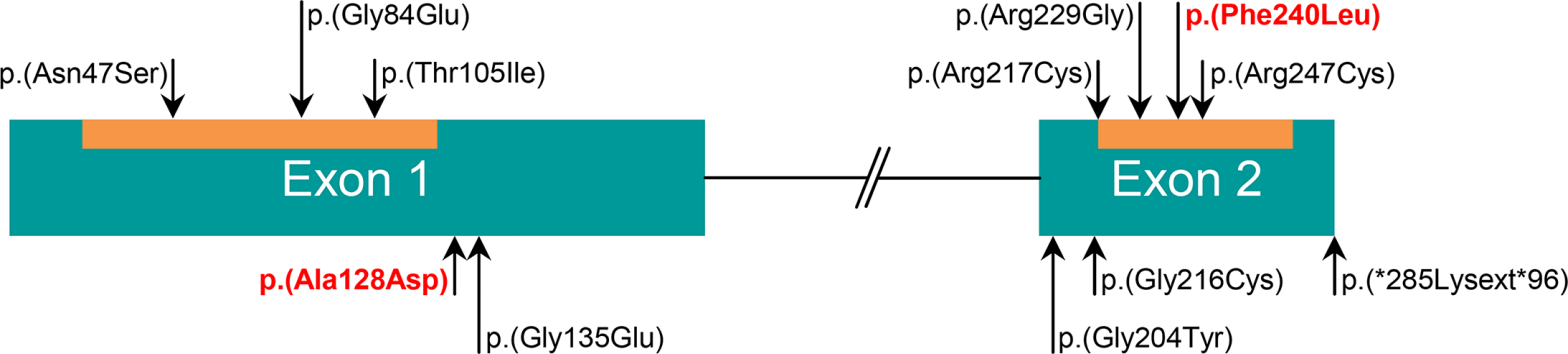
*HOXB13* structure and distribution of coding non-synonymous variants reported in prostate cancer patients. The homeobox protein Hox1A3 N-terminal domain (PF12284) and the homeobox domain (PF00046) are represented as an orange box inside each of the corresponding exons (homeobox protein Hox1A3 N-terminal domain: residues 21–123; homeobox domain: residues 217–273). Variants located within these domains are shown above the corresponding domain. Both missense variants found in our patients [p.(Ala128Asp) and p.(Phe240Leu)] are shown in red. Variants described by other authors are shown in black.

Given the predominance of the G84E mutation in Northern Europe and the lack of data on southern European populations, we have chosen to perform full sequencing of the coding region of the *HOXB13* gene instead of genotyping only for the G84E variant. In our analysis of a series of 462 index patients with early-onset and/or familial/hereditary PrCa and of a consecutive series of 178 prostate carcinomas from patients unselected for family history or age at onset, we were able to detect, in three different patients, two novel germline alterations in heterozygosity: p.(Ala128Asp) and p.(Phe240Leu). The two novel missense variants are predicted to be deleterious by several different *in silico* tools. In fact, comparing the *in silico* analyses of the two novel missense variants we here report with those of the G84E, there are no significant differences in pathogenicity scores: PolyPhen2, PROVEAN, SIFT and MutationTaster predict all three variants to be pathogenic; MutationAssessor predicts functional impact combined scores of 2.5, 2.5 and 2.9 for G84E, p.(Ala128Asp) and p.(Phe240Leu), respectively; the probabilities of the variants being deleterious calculated by MutPred are 24%, 24% and 60% for the G84E, p.(Ala128Asp) and p.(Phe240Leu), respectively. The p.(Phe240Leu) alteration is located in the highly conserved homeobox domain (Fig 4) and the predicted amino acid change occurs in a highly conserved residue (Fig 3). Even though the p.(Ala128Asp) is not located in any known domain (Fig 4), it occurs in a highly conserved amino acid (Fig 3) and highly conserved nucleotide (PhyloP: 0.89). Interestingly, genotyping data of polymorphic microsatellite markers revealed that the two subjects carrying the *HOXB13* c.383C>A germline mutation share the same allele for five markers, but not for the marker closest to the HOXB13 (D17S1326). This does not necessarily exclude a common ancestor, as recombination or mutational events could account for haplotype divergence from a single ancestor.

Several authors have not found a significant association between disease aggressiveness (namely presenting PSA, Gleason score, TNM stage, NCCN risk group, extra-prostatic disease at diagnosis, biochemical failure following treatment) and G84E carrier status [12–14,37,38], whilst Laitinen and collaborators have reported an association between the G84E mutation and a high PSA level (≥20ng/ml) at diagnosis. Furthermore, it is still not clear if G84E carriers also have an increased risk for other types of cancer in addition to PrCa. Two publications suggest that G84E mutation carriers have an increased breast cancer risk [17,41], while a larger study did not identify an association between G84E and breast cancer [42]. Regarding the association between the G84E mutation and colorectal cancer risk, no statistical significance was found in two reports [17,41], whereas another study with a wider cohort suggested an increased colorectal cancer risk [43]. A recent study suggests that the G84E mutation is associated with an increased risk for several cancer types, namely breast and bladder. Furthermore, a stronger association with the G84E mutation was found not only in patients diagnosed with multiple cancers (compared to those with any single cancer), but also in patients with PrCa diagnosis plus an additional type of cancer (compared to PrCa alone) [44]. In our study, the three patients harboring *HOXB13* missense variants presented either with intermediate- or high-risk disease and one of the three probands presented three different primary tumors, namely bladder and stomach cancer in addition to PrCa. Larger case-control studies are needed to determine if germline *HOXB13* mutations predispose to a more aggressive disease and to evaluate if they significantly increase the risk of other cancers. The answers to these questions will be crucial for correct genetic counseling and clinical care of *HOXB13* mutation carriers, since they will determine what kind of screening, treatment and follow-up we should offer to these patients and their affected or healthy relatives.

We found no evidence that somatic *HOXB13* mutations are common in prostate carcinogenesis, something that is compatible with the data in the Catalogue of Somatic Mutations in Cancer (COSMIC, http://cancer.sanger.ac.uk/cosmic/, accessed on May 15^th^ 2015) showing only two mutations in 521 prostate carcinomas tested. In fact, the only mutation we found in the 178 prostate carcinomas turned out to be a germline mutation. Interestingly, the mutation we found in the tumor was heterozygous, as indeed was the case in the only G84E carrier previously evaluated for loss of heterozygosity in the tumor [12]. Moreover, tumors of G84E carriers seem to maintain HOXB13 expression [12,17]. These two observations, associated with the fact that no truncating mutations have so far been reported in PrCa patients, are compatible with an activating oncogenic role of these missense *HOXB13* mutations. However, other authors have suggested that *HOXB13* functions as a tumor suppressor gene in prostate and other types of cancer [45–47], presumably through haploinsufficiency. The question of whether this gene is a tumor suppressor gene or a proto-oncogene is therefore still open and functional studies are needed to clarify the issue. In fact, the function of HOXB13 may depend on the cellular context, as it has been shown that it is a cell growth suppressor by inhibition of androgen-mediating signals [48], but on the other hand it may also be involved in androgen-independent survival of PrCa cells through upregulation of E2F [49].

The finding of two novel germline mutations, including two patients with the same missense mutation p.(Ala128Asp), but not the previously described G84E variant, suggests that there is geographic heterogeneity concerning the pattern of *HOXB13* mutations in early-onset or familial/hereditary PrCa and that specific testing for the G84E in populations other than those with northern European origin may not be indicated. Instead, full sequencing of the *HOXB13* coding region should be performed until the mutational pattern of each population is established.

## Supporting Information

S1 Fig Patients P308T and HPC311 microsatellite marker haplotypes. Capillary electrophoresis pattern of microsatellite markers D17S1326, D17S1323, D17S855 and D17S800 (from left to right) for patient P308T (A) and patient HPC311 (B) who carry the c.383C>A *HOXB13* mutation. Although we could not phase a haplotype for these two individuals, they present alleles in common (as indicated by the arrow), which can be compatible with a shared haplotype.
(TIF)

S1 Table Primers used for sequencing the entire coding region of the *HOXB13* gene and for KASPar SNP genotyping. (DOCX)

S2 Table Microsatellite markers genomic location, primer sequences and haplotypes of the two patients presenting the c.383C>A *HOXB13* mutation. (DOCX)
