# Supplementary material for: Identification of Two Novel HOXB13 Germline Mutations in Portuguese Prostate Cancer Patients
Source: PLoS One. 2015 Jul 15;10(7):e0132728. doi: 10.1371/journal.pone.0132728 (PMC4503425; doi:10.1371/journal.pone.0132728)
Supplement: S1 Table — (DOCX) [file pone.0132728.s002.docx]

|  | **Primer name** | **Sequence 5’→ 3’** |
| --- | --- | --- |
| *HOXB13* sequencing primers | Exon 1 forward primer | CGAGCTGGGAGCGATTTA |
|  | Exon 1 reverse primer | AGCTCCAAGTCTCCCTCCTC |
|  | Exon 2 forward primer | TTGCACGTGCGCCTGTAGGG |
|  | Exon 2 reverse primer | GTCTCCCCAGGACACCCCCA |
| *HOXB13* KASPar primers | HOXB13 c.383 C>A C allele | GAAGGTGACCAAGTTCATGCTCCCGGATATCCCGGATAGAAGG |
|  | HOXB13 c.383 C>A A allele | GAAGGTCGGAGTCAACGGATTTCCCGGATATCCCGGATAGAAGT |
|  | HOXB13 c.383 C>A common primer | TCCCACGGCCGGGGAAGAGTA |
|  | HOXB13 c.720 C>A C allele | GAAGGTGACCAAGTTCATGCTCGCCTCTTGTCCTTGGTGATG |
|  | HOXB13 c.720 C>A A allele | GAAGGTCGGAGTCAACGGATTCGCCTCTTGTCCTTGGTGATT |
|  | HOXB13 c.720 C>A common primer | CAAGAAACGCATTCCGTACAGC |
