# Supplementary material for: Identification of Two Novel HOXB13 Germline Mutations in Portuguese Prostate Cancer Patients
Source: PLoS One. 2015 Jul 15;10(7):e0132728. doi: 10.1371/journal.pone.0132728 (PMC4503425; doi:10.1371/journal.pone.0132728)
Supplement: S2 Table — (DOCX) [file pone.0132728.s003.docx]

**S2 Table. Microsatellite markers genomic location, primer sequences and haplotypes of the two patients presenting the c.383C>A *HOXB13* mutation.**

| **Markers^a^** | **Location^b^** | **Primer sequences** | **P308T^c^** | **HPC311** |
| --- | --- | --- | --- | --- |
| D2S391 | 46411503-46411648 | F- ATGGAGCCAGTAGGTTACAGC  R- GGTGAGAGGGTATGATGGAA | 140/142 | 142/146 |
| D3S3527 | 39345373-39345475 | F- ATCGGCCATATGTTGGTA  R- GGTCTGAGAAGATACTTGAT | 94/104 | 102/106 |
| D5S404 | 116847199-116847380 | F- CTGGAGATGTAATGCTGTGC  R- GATCACCACATTCCACCTAAT | 182/186 | 182/188 |
| D9S156 | 16244181-16244313 | F- ATCACTTTTAACTGAGGCGG  R- AGATGGTGGTGAATAGAGGG | 128/130 | 142/142 |
| D13S267 | 34264163-34264386 | F- GGCCTGAAAGGTATCCTC  R- TCCCACCATAAGCACAAG | 144/152 | 150/156 |
|  |  |  |  |  |
| *Cen* |  |  |  |  |
| THRA | 38240189-38241058 | F- CTGCGCTTTGCACTATTGGG  R- CGGGCAGCATAGCATTGCCT | **165**/165 | **165**/165 |
| D17S800 | 39056423-39056594 | F- GGTCTCATCCATCAGGTTTT  R- ATAGACTGTGTACTGGGCATTGA | **167**/169 | **167**/173 |
| D17S855 | 41204744-41204894 | F- GGATGGCCTTTTAGAAAGTGG  R- ACACAGACTTGTCCTACTGCC | 139/**147** | 143/**147** |
| D17S1323 | 41238044-41238193 | F- TAGGAGATGGATTATTGGTG  R- AAGCAACTTTGCAATGAGTG | **151**/153 | **151**/151 |
| *BRCA1* | 41196312-41277500 |  |  |  |
| D17S1327 | 41375460-41375591 | F- CTAAGGAGGTTTCTCTGGAC  R- TTCACAACTCAAGGTAAGATAGG | **129**/129 | **129**/129 |
| D17S1326 | 41385105-41385206 | F- CGGCCTTGCAGCTGATATTT  R- ATCGCTTGAATGTGGGAGGC | 182/182 | 180/184 |
| *HOXB13* | 46802125-46806111 |  |  |  |
| *Tel* |  |  |  |  |

^a^The common alleles in chromosome 17 are indicated in bold.

^b^Genome coordinates were derived from the National Center for Biotechnology Information (NCBI) Map Viewer (GRCh37).

^c^Haplotype of this patient was performed in DNA from peripheral leukocytes and not in tumor tissue.
